# Pain Medication Use and Perceived Effectiveness in People With Multiple Sclerosis: A Mixed‐Methods Study

**DOI:** 10.1155/prm/9981770

**Published:** 2026-07-29

**Authors:** Mohammed Suleiman Obsa, Alice Saul, Kristen Lefever, A. Julie Campbell, L. Laura Laslett, Baye Dagnew, V. Bruce Taylor, Ingrid van der Mei

**Affiliations:** ^1^ Menzies Institute for Medical Research, University of Tasmania, Hobart, Tasmania, Australia, utas.edu.au; ^2^ Department of Anaesthesia, Arsi University, Asella, Oromia, Ethiopia, arsiun.edu.et; ^3^ School of Medicine, University of Queensland, Brisbane, Queensland, Australia, health.qld.gov.au; ^4^ College of Medicine and Health Sciences, University of Gondar, Gondar, Amhara, Ethiopia, uog.edu.et

**Keywords:** epidemiology, mixed method, multiple sclerosis, pain, pain medications

## Abstract

**Background:**

Individuals with multiple sclerosis (MS) often use multiple medications, but evidence comparing their effectiveness is scarce. This study aims to (a) describe pain medication use and perceived effectiveness, (b) identify factors associated with the total number of pain medications, (c) explore the reasons for stopping and (d) use qualitative data to explore the lived experience of PwMS in relation to pain medication effectiveness and reasons for stopping.

**Methods:**

Participants from the Australian MS Longitudinal Study completed a pain survey in 2021 (*n* = 1691); 26 participants self‐nominated to participate in focus groups. Participants with regular pain were asked which pain medications they used, to what extent the medications reduced pain, which medications they stopped using and the reasons for stopping. Quantitative data were analysed using descriptive statistics; negative binomial regression was used to determine factors associated with the total number of medications. Qualitative data were analysed using thematic analysis.

**Results:**

Among 899 participants with regular pain, 87.3% were using pain medication. In terms of perceived effectiveness, 62.3% of all reported medications resulted in ‘much improvement’, ‘very much improvement’ or ‘complete pain relief’. The medications commonly reported to be effective were opioid‐containing medications, cannabinoids, antimigraine medications, a combination of paracetamol and nonsteroidal anti‐inflammatory drug (NSAID) products and sedatives. Having more pain types, greater pain severity, being female and greater disability (all *p* < 0.05) were associated with taking a higher number of medications. Common reasons for stopping a medication were side effects and ineffectiveness. Qualitative data supported the quantitative findings.

**Conclusions:**

Most people with pain used medications, and the effectiveness was acceptable for the majority of people. Despite that, stopping medications until the right ones were found was common, often due to side effects and ineffectiveness. This suggests that, with adjustments to treatment plans, effective pain control is achievable for most PwMS with pain.

## 1. Introduction

Pain is a common symptom in multiple sclerosis (MS), with a 2013 meta‐analysis finding a pooled prevalence of 63% [1]. It is one of the symptoms that most impacts health‐related quality of life [2]. The pain that people with MS (PwMS) experience may be due to: (1) the pathophysiological mechanisms of MS, which includes inflammatory demyelination and axonal damage in the central nervous system [3]; (2) secondary complications, such as musculoskeletal pain as a result of, for example, extended sitting [4]; or (3) other medical conditions (nearly 90% of PwMS experience other medical conditions [5]). Pain types that PwMS may experience include dysaesthetic extremity pain, spasticity‐associated pain, Lhermitte’s phenomenon, allodynia, trigeminal neuralgia, painful tonic spasms, headache, migraine headache, optic neuritis and musculoskeletal pain [6].

PwMS frequently use pain medications; they comprise 30% of all medications used to treat MS symptoms [7]. Classes of pain medications include paracetamol/acetaminophen, nonsteroidal anti‐inflammatory drugs (NSAIDs), opioids, antidepressants, anticonvulsants, sedatives, muscle relaxants and cannabinoids. Despite their frequent use, PwMS frequently report inadequate pain relief [8, 9] and substantial side effects [9], often leading to stopping medications [8].

There are currently no evidence‐based recommendations for pain management in PwMS. This may be partly due to the complexity with which pain presents itself in MS, with multiple pain types often present [4, 10], and limited evidence from sufficiently powered randomised controlled trials (RCTs) addressing MS‐related pain [11–15]. While many PwMS experience multiple types of pain and use several medications concurrently [9], most previous RCTs have focused on a single type of medication [16–22] or compared only a limited number of medications, targeting specific types of pain [23–30]. This may suggest that treatment effectiveness in the context of polypharmacy is often inadequately represented in RCTs, highlighting the importance of exploring patients’ perspectives in real‐world settings. However, there are only three observational studies that examined the perceived effectiveness of multiple pain medications in MS [8, 31, 32], using ranking or relief ratings [31]. All studies were conducted in a single country (the United States), with one published more than 20 years ago. Two of these studies found that opioids had the highest pain relief rating [8, 31], with one of them also finding that nonprescription medications were most commonly used, but only moderately effective [31]. These studies did not assess medication‐taking behaviour, including persistence, switching and reasons for discontinuation or switching. Understanding these factors is important, as issues like side effects, severe adverse effects and low effectiveness play an important role in pain management.

While the measured perceived effectiveness includes the ‘placebo and nocebo effect’, these observational studies are particularly helpful when comparing the effectiveness of multiple medications. A comprehensive picture of pain medication use, perceived effectiveness and reasons for stopping among PwMS may inform the design of future pain RCTs and contribute to the evidence base of future recommendations for personalised pain management.

The aim of this study was to (a) describe patterns of pain medication use and their perceived effectiveness, (b) identify the factors associated with the total number of pain medications, (c) explore the reasons for stopping and (d) supplement the quantitative findings in aims (a) and (c) by using qualitative data that explored the lived experience of PwMS in relation to pain medication effectiveness and reasons for stopping.

## 2. Methods

### 2.1. Study Design, Information Sources and Study Population

This study used a mixed‐methods approach to capitalise on the strengths of both quantitative and qualitative data within a single design. In this case, qualitative data were used to supplement the quantitative data and provide a deeper contextualisation and nuance to the quantitative data [33, 34]. For the overall study, we were guided by validated guidelines, including the Good Reporting of a Mixed Methods Study (GRAMMS) checklist (Supporting Table 1) [35]. Additionally, for the focus groups, we employed the Standards for Reporting Qualitative Research [36], a list of 21 elements considered important to reporting qualitative research in a transparent manner, as well as the Consolidated Criteria for Reporting Qualitative Data (COREQ) for focus groups and interviews (Supporting Table 2) [37]. The STROBE statement for cross‐sectional studies was used for the reporting of quantitative findings (Supporting Table 3). The integration of quantitative and qualitative data was purposefully embedded at multiple stages of the study, including the study design, at the point of study participant selection, data collection, data analysis and interpretation of findings. Integration at the interpretation stage was achieved through narrative synthesis weaving, whereby quantitative and qualitative findings were examined together to provide a comprehensive understanding of the findings.

We used data from the Australian MS Longitudinal Study (AMSLS). The AMSLS was established in 2002 [2] and currently includes over 2500 active participants. Every participant in the AMSLS provides written consent upon enrolment. The study has been approved by the Tasmanian Health and Medical Human Research Ethics Committee (H0014183). We obtained separate ethical approval for the qualitative research (H0026997) from the University of Tasmania. The study was conducted in accordance with the principles of the Declaration of Helsinki.

The quantitative data were primarily obtained from the 2021 Pain Survey (Aug–Oct 2021). Invitations were sent to 2543 individuals, with 1691 (66.5%) completing the survey. According to their preference, 1916 (75.3%) participants completed the surveys online (∼80%) using LimeSurvey, 595 (23.4%) participants completed a paper‐based questionnaire distributed by mail, and 32 (1.3%) received assistance by completing it by telephone. Paper‐based questionnaires were manually verified after being scanned by automated software, and manual verification was needed. To characterise the Pain Survey participants, several demographic and clinical variables were derived from the 2021 Medication and Disease Course Survey (Oct–Dec 2021) or other surveys, where these variables were not time‐dependent. The Pain Survey: The Pain Survey included an invitation to a focus group discussion for those with regular pain, with 198 participants expressing their interest. Of these, 26 PwMS with regular pain were involved in the focus group discussions.

### 2.2. Measurements

#### 2.2.1. Quantitative Data on Pain Medication

Participants were first asked whether they identified themselves as someone who had pain regularly (this pain could be part of their MS or due to other medical conditions). This pain could have been part of their MS or due to other medical conditions. Those with regular pain were asked whether they were using any pain medications (yes/no). It was indicated to them that these could be medications prescribed to them, medications they had purchased from the chemist or supermarket, medications given to them by a friend or family member, or supplements and alternative medications such as cannabis. In a separate section, they were asked whether there were any pain medications they used in the past but stopped using (yes/no). For both sections, if they stated yes, then participants were asked to name the pain medication(s) and classify the medication (response categories included opioid, paracetamol, NSAIDs, antidepressant, anticonvulsant, sedative, muscle relaxant, other and unsure). A list of pain medications, including the brand names, active ingredients and medication types, was provided to assist with the classification. When processing the data, all entries were checked, and new categories were developed for: antimigraine medications (any medication or supplement containing antimigraine properties), cannabinoids (any medication or supplement containing cannabis‐related products), and paracetamol and NSAIDs (for medications containing a combination of paracetamol and NSAIDs). Furthermore, combination products containing opioids as an active ingredient (e.g. opioids and paracetamol; opioids, paracetamol and sedatives; and opioids and NSAIDs) were combined with the pure opioids and labelled as ‘opioid‐containing medications’, justified due to similar perceived effectiveness in our dataset compared to pure opioid medications. We excluded disease‐modifying therapies, alternative treatment strategies and supplements.

For each pain medication used, we asked to what extent they believed the medication reduced their pain (response categories: ‘no improvement’, ‘minimal improvement’, ‘much improvement’, ‘very much improvement’, ‘complete pain relief’ and ‘unsure’). For each medication that was stopped, we asked the reasons for stopping (response categories: ‘I didn’t like the side effects’, ‘it was ineffective’, ‘it stopped being effective’, ‘it was too expensive’, ‘I didn’t need it anymore’ or ‘other reasons’).

#### 2.2.2. Qualitative Data

Qualitative data were gathered to supplement the quantitative data. We considered the principle of ‘information power’ when determining the size and composition of the focus groups [38]. Informed by this theory of information power and to enable richer discussion, we recruited five smaller focus groups within generational age groups, namely younger (18–39 years), middle‐aged (40–65 years) and older (> 65 years). PwMS in the younger age group did not volunteer to participate. Virtual focus groups were conducted using the Zoom platform, each taking no more than 1.5 h. The facilitator guide was developed by A. Julie Campbell (a health economist, researcher and experienced qualitative researcher) and Ingrid van der Mei (a professor in epidemiology), both of whom had prior experience conducting research with PwMS. At the time of the study, A. Julie Campbell conducted the focus groups, while Mohammed Suleiman Obsa (a PhD student and experienced qualitative research assistant) observed the discussions. A verbatim transcription of the audio was conducted with identifying information removed. Aligned with the quantitative survey, participants were asked to discuss the use of pain medications, the effects of their pain medications on pain and the reasons for stopping medications.

#### 2.2.3. Other Measures

We had data on age, sex, highest educational attainment, whether they were employed, MS duration since diagnosis, MS duration since first MS symptom and the patient‐determined disease steps (PDDS), a measure of disability level.

### 2.3. Data Analyses

Analyses were performed using Stata Version 17 (Stata Corp. LP, College Station, USA). We used descriptive analyses to summarise the characteristics of the study participants, the data on medication use, perceived effectiveness and reasons for stopping medications. Descriptive analyses were also used to summarise the characteristics of the study participants using means and standard deviations for normally distributed continuous variables, as well as medians and interquartile ranges for skewed continuous variables. To examine factors associated with the total number of pain medications used, negative binomial regression with a manual backward stepwise regression approach was employed due to overdispersion in the count outcome, whereby the variance (2.8) exceeded the mean (2.3).

A nonresponse bias analysis was conducted to evaluate whether there were differences in characteristics between those who completed the pain survey and those who were invited but did not complete the survey, using *t*‐tests for continuous variables and chi‐squared tests for categorical variables.

Based on validated qualitative research guidelines [36, 37], focus group data were analysed thematically with the assistance of NVivo software and triangulated with quantitative results to provide deeper contextualisation and nuance to the quantitative results [39]. Thematic analysis was conducted using both inductive/deductive approaches [40, 41], where themes emerged from both the quantitative data and the pre‐existing framework of pain medication utilisation, effectiveness of these medications for pain and reasons for stopping. MSO coded the data, after which codes and emerging themes underwent discussion among the authors, Mohammed Suleiman Obsa, A. Julie Campbell and Ingrid van der Mei.

## 3. Results

### 3.1. Demographic and Clinical Profile of Participants

Half of the respondents (53.2%, *n* = 899/1691) indicated that they had regular pain. Among those, 87.3% (782/896) were using one or more pain medications during the survey (Table 1). Compared to the total sample, those with regular pain were more likely to be female, less likely to be employed, and less likely to have a university postgraduate degree. Additionally, they were, on average, slightly older and had a longer duration since symptom onset and a higher PDDS score. Compared to those with regular pain, those also using pain medication were more likely to be employed (33.9% vs. 30.3%) and slightly more likely to be female (84.5% vs. 82.9%). The groups were comparable with regard to age, duration of symptoms since onset, duration of disease since diagnosis, educational level and PDDS score (Table 1).

**TABLE 1:** Demographic and clinical profile of participants.

Characteristics	Total sample in pain survey (*n* = 1691)	Total sample with regular pain (*n* = 899)	Total sample with regular pain who used pain medication (*n* = 782)
Female, % (*n*)	79.2 (1339)	82.9 (745)	84.5 (661)
Employed, % (*n*)	39.4 (624)	30.3 (216)	33.9 (205)
Educational level, % (*n*)			
Primary or secondary school	23.6 (399)	26.5 (238)	26.9 (207)
Occupational certificate or diploma	18.2 (567)	33.7 (303)	33.6 (258)
University bachelor’s degree	22.7 (384)	21.3 (191)	21.5 (165)
University postgraduate degree	22.8 (318)	17.0 (153)	18.0 (138)
Age in years, mean (SD)	58.6 (11.5)	59.4 (10.7)	59.5 (10.7)
Disease duration since diagnosis in years, mean (SD)	17.0 (9.1)	17.4 (9.0)	17.4 (9.1)
Disease duration since symptom onset in years, mean (SD)	21.5 (11.3)	22.5 (11.3)	22.6 (11.8)
PDDS, mean (SD)	2.7 (2.4)	3.2 (2.3)	3.3 (2.3)

*Note:* Among people with regular pain, 3 did not report whether they used pain medications and were excluded from the overall medication utilisation analysis. We had the following missing data among the total pain survey participants: 23 for educational level, 342 for employment status, 8 for disease duration since diagnosis, and for disease duration since symptom onset.

Abbreviations: PDDS, patient‐determined disease steps; SD, standard deviation.

### 3.2. Utilisation and Perceived Effectiveness of Pain Medication Types

Among those with regular pain, 13% (*n* = 118) did not use any pain medication, 23.3% (*n* = 209) used one medication, 25.4% (*n* = 228) two medications, 18.2% (*n* = 164) three medications, 9.9% (*n* = 89) four medications, and 10% (*n* = 91) five or more medications. The median of the total number of pain medications used per person was two (interquartile range 1–3).

When we examined the use of pain medications and ordered based on the number of reports, we found that the most frequently reported medication types were paracetamol, NSAIDs, opioid‐containing medications, anticonvulsants, antidepressants and muscle relaxants. In contrast, cannabinoids, sedatives, antimigraine medications and medications containing a combination of paracetamol and NSAIDs were less frequently used. When we examined the use of pain medications based on the number of people, we found results in a similar order to those obtained based on the number of reports (Table 2).

**TABLE 2:** Utilisation and perceived effectiveness of pain medications by type among those with regular pain who used pain medications.



*Note:* Pain medication utilisation was categorised by the number of reports and people. They were ranked from highest to lowest percentage of utilisation. The percentage of pain medication utilisation was done across column. A total of 0.3% (*n* = 3/899) did not report whether they used pain medications and were excluded from the overall medication utilisation analysis. A total of 2.2% (*n* = 46/2052) of reports on medication used did not indicate its perceived effectiveness and were excluded from effectiveness analysis. NSAIDs: nonsteroidal anti‐inflammatory drugs. Opioid∗ represents pure opioids (156), opioid + paracetamol (76), opioid + paracetamol + sedative (19) and opioid + NSAIDs (17) by the number of reports and pure opioids (127), opioid + paracetamol (73), opioid + paracetamol + sedative (19) and opioid + NSAID (17) by the number of people. Paracetamol + NSAIDs∗∗ represents medications containing a combination of paracetamol and NSAIDs. Other pain medications included were as follows: Actonel (1), Alcohol (1), Aminopyridine (1), Arthro‐Aid Direct (1), Australian Karma Rub (1), Cadivast (1), Capsaicin (1), Cordilox (2), De‐gas (1), Dencorub (4), Eleuphrat (1), Fampridine (3), Goanna anti‐inflammatory cream (1), Hydroxychloroquine Plaquenil (4), Ice gel (1), Inflavonoid Intensive Care (1), Infliximab (1), Immunoglobulin infusion (1), (Levodopa (1), Madopar (2), Melatonin (1), Misoprostol (1), Neurogenic (1), Neurogenic plus (1), Nexium (4), Nitrostat (1), Oxybutynin (1), (Pantoprazole (1), Perindopril (1), Pramipexole (2), Progout (1), Rabeprazole (1), Rennie (1), Ritalin (1), Sifrol (7), Simipex (1), Sinemet (2), Somac (1), Systane Balance (1), Tiger Balm (2), Versatis patch (1), Vita pos (1), Zometa (1), Zostrix (1) and Zoton (1).

**FIGURE 1:**
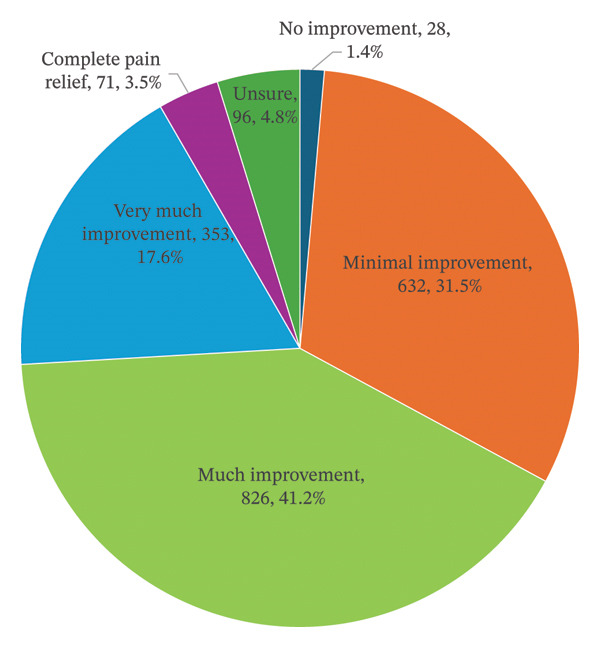
Perceived effectiveness of pain medications when all medications combined (*n* = 2006 reports of medications on pain reduction).

In terms of perceived effectiveness, 62.3% (*n* = 1250/2006) of all reported medications resulted in much improvement, very much improvement or complete pain relief (Figure 1). The medication types most frequently reported to be effective included cannabinoids, opioid‐containing medications, antimigraine medications, medications containing a combination of paracetamol and NSAIDs, and sedatives (all > 70%) (Table 2). Lower effectiveness was reported for anticonvulsants, NSAIDs alone, antidepressants, muscle relaxants and paracetamol (between 50% and 67%), despite some of these being the most frequently used.

### 3.3. Factors Associated With the Total Number of Medications Used

In the multivariable analysis, factors associated with taking a higher number of pain medications included having a greater number of pain types, greater pain severity, being female and greater PDDS (all *p* < 0.05) (Table 3).

**TABLE 3:** Factors associated with the total number of pain medications used among PwMS with regular pain.

Characteristics	Univariable analysis		Multivariable analysis	
	*β* (95% CI)	*p* value	*β* (95% CI)	*p* value
Total number of paint types	0.40 (1.45, 1.53)	**< 0.01**	0.10 (0.08, 0.13)	**< 0.01**
Overall pain severity (0–10)	0.08 (1.06, 1.11)	**< 0.01**	0.06 (0.03, 0.09)	**< 0.01**
Female	0.43 (1.26, 1.86)	**< 0.01**	0.17 (0.01, 0.33)	**0.04**
PDDS	0.14 (1.11, 1.18)	**< 0.01**	0.02 (0.00, 0.05)	**0.05**
Disease duration since symptom onset	0.01 (0.00, 1.01)	**0.04**	NR	NR
Education status				
Primary or secondary school	(Reference)			
Occupational certificate or diploma	−0.07 (−0.24, 0.10)	0.41		
University bachelor’s degree	−0.15 (−0.35, 0.04)	0.13		
University postgraduate degree	−0.25 (−0.45, −0.05)	0.01		
Age	0.00 (0.99, 1.01)	0.28		
Type of MS				
Primary progressive MS	(Reference)			
Relapsing remitting MS	−0.01 (0.31, 1.11)	0.36		
Secondary progressive MS	0.13 (−0.11, 0.38)	0.29		
Progressive relapsing MS	0.26 (−0.19, 0.71)	0.26		
Unsure	−0.48 (−0.81, −0.15)	**< 0.01**		

*Note:* A *p* value of less than 0.05 was bolded in the analysis. PDDS stands for patient‐determined disease steps. The ‘Other’ category of educational status (*n* = 2) was excluded from the univariable analysis due to small sample size and lack of analytical relevance. NR—not retained in the multivariable analysis as it was no longer significant. Duration of symptoms since onset was excluded from the multivariable analysis in the first stage, as it was not associated with the initial stage of backward stepwise regression analysis (*p* = 0.242). Educational level was also excluded in the second stage, as all categories of education levels were not associated with the total number of pain medication uses (*p* > 0.05).

### 3.4. Pain Medications That Were Stopped and Reasons for Stopping

Among those with regular pain, 40.2% (*n* = 353/878) of participants had stopped one or more medications. The 353 participants reported a total of 641 medications that they had stopped. Among these, the pain medication types most frequently stopped were opioid‐containing medications (27.6%), anticonvulsants (25.6%) and NSAIDs (11.9%) (Table 4).

**TABLE 4:** Reasons for stopping past pain medications in those with regular pain in the AMSLS.

Pain medication type	Total reports of medications (*n* = 641)	I did not like side effect (*n* = 239)	It was ineffective (*n* = 129)	It stopped being effective (*n* = 39)	It was too expensive (*n* = 11)	I did not need it anymore (*n* = 89)	Other (*n* = 128)	Reason for other
	% column (*n*)	% row (*n*)						
Opioid∗	27.6 (177)	**37.9 (67)**	11.9 (21)	5.6 (10)	0.5 (1)	19.8 (35)	24.3 (43)	Instructed by professional to stop (13), (adverse effects (6), government restriction or legality (6), unavailability (5), still in use with reduced frequency or dose (4), replaced with another medication (3), I didn’t like the side effects, and it was ineffective (2), ran out or I used it all (1), I didn’t like side effects and didn’t need it anymore (1), It was too expensive and it was ineffective (1) and unspecified (1).
Anticonvulsant	25.6 (164)	**50.0 (81)**	25.3 (41)	8.6 (14)	0.0 (0)	7.4 (12)	8.6 (14)	I didn’t like the side effects, and it was ineffective (5), adverse effects (5), still in use with reduced frequency or dose (4), I didn’t like side effects and didn’t need it anymore (1) and replaced with another medication (1).
NSAIDs	11.9 (76)	23.7 (18)	22.3 (17)	1.3 (1)	1.3 (1)	22.3 (17)	**29.0 (22)**	Adverse effects (8), instructed by professionals to stop (6), replaced with another medication (3), still in use with reduced frequency or dose (3), I didn’t like side effects and didn’t need it anymore (1) and drug‐on‐drug interaction (1).
Antidepressant	7.3 (47)	**48.9 (23)**	17.0 (8)	6.4 (3)	0.0 (0)	6.4 (3)	21.3 (10)	I didn’t like the side effects, and it was ineffective (3), adverse effects (3), replaced with another medication (1), unavailability (1), drug‐on‐drug interaction (1), and I didn’t like the side effects and it was stopped being effective (1).
Cannabinoid	7.0 (45)	28.9 (13)	15.6 (7)	6.7 (3)	13.3 (6)	2.2 (1)	**33.3 (15)**	I didn’t like the side effects, and it was ineffective (3), government restriction or legality (3), unavailability (3), still in use with reduced frequency or dose (2), It was too expensive and it was ineffective (1), run out or I used it all (1), replaced with another medication (1) and unspecified (1).
Paracetamol	6.5 (42)	14.3 (6)	**42.9 (18)**	4.8 (2)	2.4 (1)	26.1 (11)	9.5 (4)	Adverse effects (1), unavailability (1), replaced with another medication (1) and drug‐on‐drug interaction (1).
Muscle relaxant	3.3 (21)	**38.1 (8)**	3.3 (7)	9.5 (2)	0.0 (0)	0.0 (0)	9.1 (4)	Adverse effects (2), I didn’t like the side effects, and it was ineffective (1) and 1 (run out or I used it all (1).
Sedative	3.0 (19)	**66.7 (12)**	0.0 (0)	0.0 (0)	0.0 (0)	5.6 (1)	27.8 (5)	Still in use with reduced frequency or dose (2), I didn’t like the side effects, and it was ineffective (1), government restriction or legality (1) and replaced with another medication (1).
Antimigraine medications	2.5 (16)	25.0 (4)	12.5 (2)	6.2 (1)	0.0 (0)	18.8 (3)	**37.5 (6)**	I didn’t like the side effects, and it was ineffective (2), adverse effects (1), still in use with reduced frequency or dose (1), instructed by professional to stop (1) and unavailability (1)
Paracetamol + NSAIDs∗∗	1 (0.2)	0.0 (0)	0.0 (0)	0.0 (0)	0.0 (0)	0.0 (0)	1 (100.0)	Adverse effects (1)
Other	2.6 (17)	6.3 (1)	**31.3 (5)**	18.7 (3)	6.3 (1)	18.7 (3)	18.7 (3)	Adverse effects (2), unavailability (1)
Unsure	2.5 (16)	**42.9 (6)**	21.4 (3)	0.0 (0)	7.1 (1)	21.4 (3)	7.1 (1)	1 (adverse effects).

*Note:* Pain medication types are ranked based on the medication most frequently stopped, with bolded values representing the most common reason for stopping. A total of 2.3% (*n* = 21/899) of participants did not report whether they stopped using medications and were excluded from this analysis. For 0.9% (*n* = 6/641) reports of pain medications stopped, the reason for stopping was not specified and therefore considered missing when reporting the reasons for stopping. Opioid∗ represents opioid‐containing medication, with reports of pure opioids (132), opioid + paracetamol (37), opioid + paracetamol + sedative (6) and opioid + NSAIDs (2). Paracetamol + NSAIDs∗∗ represents medications containing a combination of paracetamol and NSAID. NSAID: nonsteroidal anti‐inflammatory drug. Other pain medications included were as follows: capsaicin (2), Dencorub (2), Dytrex (1), Pramipexole (1), Rotigotine (1), Salome (1), Sifrol (1), Alcohol (1), Aminopyridine (1), Arava (2), Ice Gel (1), Mobilis (1), Neu (1) and Cadivast (1).

Out of 641 reports of medication stopped, 635 indicated reasons for stopping. The most frequently reported reasons included side effects (37.6%) and perceived ineffectiveness (20.3%). The pain medication types most frequently stopped due to side effects included sedatives (66.7%), anticonvulsants (50.0%), antidepressants (48.9%), muscle relaxants (38.1%) and opioid‐containing medications (37.9%). The pain medication type most frequently stopped due to perceived ineffectiveness was paracetamol (41.9%). It was also reported that the pain medication type most frequently stopped due to being expensive was cannabinoids (13.3%).

### 3.5. Potential Selection Bias and Effects on Key Findings

We compared 2021 pain survey respondents and those who were invited but did not respond on key demographic and clinical factors. Respondents were slightly older than nonresponders (+2.0 years, *p* < 0.01), while there were no differences by sex, educational status, duration of MS and MS symptoms.

To examine whether having a slightly older sample affected our key findings, we stratified our sample by the median age (59 years). In terms of overall medication use, there was no significant difference in prevalence estimate (above median age: 88.1%; below median age: 86.3%; *p* = 0.43). Similarly, there was little difference in the perceived effectiveness of all medications (above the median age: 64.4% reported much improvement, very much improvement or complete pain relief; below the median: 60.5%; *p* = 0.146). However, older participants were less likely to stop using pain medications (above the median: 36.2%; below the median: 42.7%; *p* = 0.05).

### 3.6. Composition of Focus Groups

Twenty‐six participants were recruited to five focus groups that mainly comprised females (24, 92.3%) with a mean (SD) age of 61.5 years (6.71). There were two focus groups in the older age category (focus Group 1 [FG1]: 4 females, 1 male, mean age 69.8 years; focus Group 4 [FG4]: 5 females, mean age 67.6 years). There were three middle‐aged focus groups (focus Group 2 [FG2]: 4 females, mean age 58.75 years; focus Group 3 [FG3]: 5 females, 1 male, mean age of 57.33 years; focus Group 5 [FG5]: 6 females, mean age of 55.5 years). There was a similar representation across the Australian States’ capital cities (*n* = 15) and regional and remote areas (*n* = 11), as per the Australian Statistical Geographical Standard [42].

### 3.7. Thematic Analysis

Table 5 reveals a summary of the codes, subthemes and core themes that emerged from our thematic analysis. Table 6 supports Table 5 with examples of verbatim quotes for each of the subthemes. Two main themes were identified for PwMS with regular pain, namely: (1) the perceived effectiveness of pain medications and (2) the side effects or adverse events of pain medications. Overall, this study demonstrated the substantial effects of pain medication, with many participants describing moderate or substantial pain improvement. The reduction in pain allowed some participants to ‘go to sleep that night’, indicating improved sleep. However, the qualitative data also revealed that some people stopped taking pain medications because of their side effects or adverse events. For example, one participant indicated that he could not take any ‘nerve medications’ that health professionals had suggested because ‘they tend to just space me out all the time’. This example shows the challenges of pain management, where the benefits of pain relief need to outweigh the side effects.

**TABLE 5:** Data abstraction process and resulting main theme and subtheme.

Main theme	Subtheme	Codes by pain medication type)
Theme 1: perceived effectiveness of pain medication on pain reduction (*n* = 31 comments).	Substantial improvement (*n* = 14 comments)	‐ Cannabis oil (2) ‐ Baclofen (2) ‐ Botox (3) ‐ Multiple pain medications (2) ‐ Norspan patch (1) ‐ Ampyra (1) ‐ Lyrica (1) ‐ Gabapentin (1) ‐ Endep (1)
	Moderate improvement (*n* = 9 comments)	‐ Cannabis oil (3) ‐ Multiple pain medications (2) ‐ Amitriptyline (1) ‐ Tapentadol (1) ‐ Anticonvulsants (1) ‐ Targin (1)
	Minimal improvement (*n* = 8 comments)	‐ Cannabis oil (2) ‐ Multiple pain medications (4) ‐ Amitriptyline (1) ‐ Name not specified (1)

Theme 2: pain medication side effects or adverse effects (*n* = 13)	Stopped due to side effects (*n* = 6 comments)	‐ Multiple pain medications (6) ‐ Lyrica (1) ‐ Amitriptyline (1)
	Continued and managed side effects (*n* = 3 comments)	‐ Multiple pain medications (2) ‐ Botox (1)
	Did not use pain medications at all due to fear of addiction (*n* = 2 comments)	‐ Opioid (2)
	Stopped due to adverse events (*n* = 2 comments)	‐ Multiple pain medications (1) ‐ Tegretol (1)

*Note:* If the participant reported two or more pain medications, it was stated as ‘multiple pain medications’. If the participant was uncertain about the name of a pain medication, it was recorded as ‘name not specified’.

**TABLE 6:** Subtheme and examples of verbatim quotes from the five focus groups regarding the lived experiences of using pain medication among PwMS with regular pain.

Subtheme	Example quotes
*Theme 1: perceived effectiveness of pain medication on pain reduction*	
Substantial improvement	‘I think the **baclofen** actually stops the spasms. And that’s really helpful. Yeah. And the **aminopyridine** that I take, the – for the foot drop, actually helps the functioning of my leg much better…’ FG4, female, older‐aged group).
	‘…So, my resolution was to take the **cannabis** oil. And I take that at night, and I get a good five, 6 hours sleep, solid, before I have to get up and go to the bathroom…’(FG2, middle‐aged group, Female 3)
	‘…I try **gabapentin** for the pain. And that was effective probably for a couple of months before it started to just wear off…’ (FG2, middle‐aged groups, Female 3)

Moderate improvement	‘…I’ve in the last 6 months got onto medicinal **cannabis**. Mostly that allows me to get 5 hours’ sleep not necessarily altogether but broken up…’ (FG4, older‐aged group, Female 1)
	‘Well, I’m on **tapentadol** at the moment for my shoulder, and all it’s done is taken it away so that I can actually sleep. It doesn’t completely go’. (FG5, middle‐aged group, Female 2)

Minimal improvement	‘…I’m still waiting to see whether the **amitriptyline** does anything or not. Yeah, it’s varied. I think maybe it just dulls things a bit so that you can manage a bit better, rather than making it go away’. (FG5, middle‐aged group, Female 3)

*Theme 2: pain medication side effects or adverse effects*	
Stopped using due to side effects	‘…The **Lyrica**. But of course, I can’t stay on that. I gain weight and my blood pressure goes sky high if I’m on it for too long’. (FG4, older‐aged group, Female 1)

Avoidance of pain medication use due to fear of addiction	‘…The only thing they can say to me is, “I can give you **opioids**.” I don’t want **opioids** because I don’t want to get on that track of taking tablets that may make me addicted. I don’t want that. I have enough to deal with in my life without dealing with addiction’, (FG1, older‐aged group, Female 1)

Continued using despite side effects	‘…I get **Botox injections**, so my mouth’s crooked, which I don’t care about. But that helps a lot. But when it’s in its worse stage I have to take all the pills with it. But that’s every 10 weeks that’s’ (FG4, middle‐aged group, Female 3)

Stopped using due to adverse effects	‘…Yeah. I’ve tried a lot of stuff, and I found that when you have to look holistically at your body, what it does – so they did **Tegretol** for a while, and my liver wanted to roll over and play dead’. (FG5, older‐aged group, Female 4)

*Note:* The specific name of medications was bolded to provide more emphasis.

The core themes elicited from the qualitative data are explored in further detail below.

#### 3.7.1. Theme 1: Perceived Effectiveness of Pain Medication

Within the core theme of perceived effectiveness of pain medications, three subthemes emerged that were coded by the level of improvement in MS‐related pain severity: (1) substantial improvement, (2) moderate improvement and (3) minimal improvement. Overall, this study’s qualitative data revealed meaningful effects of pain medication on pain management for PwMS, with most of the comments indicating substantial or moderate pain improvements. For example, some PwMS mentioned that the pain medications ‘helped me enormously in giving me some rest’ or that the medications ‘helped with spasms’. Furthermore, regarding the subtheme of ‘substantial improvement’ or ‘moderate improvement’, participants described pain improvement in three distinct ways: (1) improvement following medication use without specifying the type of pain, (2) improvement after using medication for a specific pain type and (3) improvement that lasted only for a limited duration. For example, one participant who used Botox injections reported substantial pain improvement without specifying the type of pain or duration of effectiveness:

“… I’ve been for probably six, seven years now going every ten weeks to my neurologist and I get Botox injections … that helps a lot.” (FG4, middle‐aged group, Female 3)

In contrast, other participants described ‘substantial improvement’ or ‘moderate improvement’ in relation to specific pain types. For example, participants who used baclofen, cannabis oil and buprenorphine patch (Norspan patches) indicated significant improvement in spasticity‐associated pain rather than neuropathic pain.

“I’m on a NORSPAN patch, originally prescribed for my neuropathic pain but I found that it does significantly help with the spasms…” (FG1, older‐aged group, Female 5).

“I was put on medicinal cannabis a few years ago…I’m on a very low dose. But it does help a bit with the spasticity, but it doesn’t touch the pain. The neuropathic pain.” (FG1, older‐aged group, Female 5)

“I think for me, I have had that every now and then I get this spasm in my leg. And it’s just as though the leg won’t relax … So, I just have the baclofen there and then I can go to sleep that night…” (FG5, middle‐aged group, Female 6)

Some participants also indicated that the ‘substantial improvement’ or ‘moderate improvement’ they experienced following medication use may decrease over time.

“…I try gabapentin for the pain. And that was effective probably for a couple of months before it started to just wear off…” FG2, middle‐age group, Female 3)

“And when you talk about managing allodynia or managing spasms or managing depression, a lot of the medications on offer are not just singularly for one thing. So, you might have an antidepressant that you’re actually being given for pain…And many of them you try along the lines, they have life‐changing effects for a short time…”

Some participants reported that pain medications resulted in only minimal pain improvement. One person said that

“…, I′ve obviously tried Panadol and ibuprofen and all of those things…they don’t really help unless it’s just a standard headache or something that’s not neurological pain, I feel.” (FG3, middle‐aged group, Female 3)

#### 3.7.2. Theme 2: Pain Medication Side Effects and Adverse Events

Within the theme of pain medication side effects and adverse events, three subthemes emerged that were coded based on the safety profile. These included side effects or adverse events that led to the medication stopping, avoidance of medication use due to fear of addiction and continued use despite side effects. Most comments in this theme discussed side effects, with some described as adverse events, as they were severe and uncommon. Overall, the data suggest that side effects are common and impact the decision to continue or stop using medications. For example, one participant who received Botox injections reported the feeling of ‘mouth’s crooked’ but continued to use it as it helped a lot with pain reduction. In contrast, another participant who used pregabalin (Lyrica) reported stopping this medication due to its side effects. Specifically, one participant said that:

“…The Lyrica. But of course, I can’t stay on that. I gain weight and my blood pressure goes sky high if I’m on it for too long.” (FG4, older aged group, Female 1)

Another participant stated that they did not use the offered medication due to fear of addiction.

“…The only thing they can say to me is, “I can give you opioids.” I don’t want opioids because I don’t want to get on that track of taking tablets that may make me addicted…” (FG1, older‐age group, Female 1)

Another participant who used carbamazepine (Tegretol), pregabalin (Lyrica) and amitriptyline stopped using them after observing adverse events:

“Yeah. It’s just that the adverse reactions you get from these medications is sometimes greater, physically and with all your internal organs as well, more damaging than the pain.” (FG5, middle aged group, Female 3)

## 4. Discussion

This is the first large cohort mixed‐methods study that examined the use and effectiveness of pain medications for PwMS in a real‐world setting. The majority of PwMS who had pain regularly used medications, with 62.3% of medication reports resulting in much improvement, very much improvement or complete pain relief. The most effective medication types were cannabinoids, opioid‐containing medications, antimigraine medications, medications containing both paracetamol and NSAIDs, and sedatives. Those more likely to use greater numbers of medications were females, those reporting more pain types, greater overall pain intensity and a greater disability level. Medications were frequently stopped, with common reasons including side effects (37.6%) and perceived ineffectiveness (20.3%). Qualitative results corroborated quantitative findings, with many comments indicating moderate to substantial pain improvement and side effects as reasons for stopping medications.

Pain medication use was common among PwMS, with as many as 87.3% of PwMS with regular pain using at least one pain medication, and 63.5% were using two or more pain medications. This aligns with another study, which focused on prescription medications only and found that 73% of PwMS with pain used prescription medications [43]. Our study is novel in its ability to facilitate comparisons between pain medication types. We found that the most frequently used pain medication types are paracetamol, NSAIDs, opioid‐containing medications, anticonvulsants and antidepressants (10%–25% of reports; 25.1%–62.4% of participants). Less frequently used pain medications included antimigraine medications, muscle relaxants, cannabinoids and sedatives. The high use of paracetamol and NSAIDs is likely due to their availability without prescription because of their lower side effect profile, which facilitates easy access for PwMS. In contrast, prescription medications, including opioid‐containing medications and cannabinoids, are less commonly used as these are not available over‐the‐counter, and their prescription is monitored as they are associated with physical and psychological dependence. Other studies reported a prevalence of opioid use in the range of 12.2%–37.7% [22, 31, 44]; in our study, 27.0% were using opioid‐containing medications, including 16.2% using pure opioids. Variations in opioid prescribing practices may reflect differences in prescription regulations, clinical guidelines and public health concerns.

In our study, we found that females, those with more pain types, those with greater overall pain severity and those with greater disability severity used a greater number of pain medications. Previous studies also observed that those with greater pain severity more frequently use pain medications [45–47]. We found that the majority used multiple pain medications, either because different pain types required different medications or because the pain was not controlled with a single medication. The latter follows the principle of multimodal analgesia, which hypothesises that using two or more pain medications improves pain relief more than using a single pain medication alone, as it targets multiple pain pathways [48]. It is important for healthcare practitioners to consider how different medications may work together to achieve the desired effects, while also managing the risks that may be associated with drug–drug interactions [49, 50]. Additionally, it will be helpful for PwMS to be educated about the possible need for using multiple medications.

Importantly, we found a relatively high perceived effectiveness, with 62.3% of all medication reports indicating a much improvement, very much improvement or complete pain relief. This finding was corroborated by the qualitative data, with most of the comments indicating moderate or substantial pain improvements. Our findings are in contrast to previous studies, indicating patients reporting inadequate pain relief [51, 52]. The higher effectiveness in our study can be partially attributed to the self‐selection of medications, whereby people continue the medications that work while they discontinue those that don’t work, either because they were not effective, stopped being effective or resulted in side effects or adverse events (see below). Importantly, our findings suggest that eventually most patients are satisfied with their pain management regimen and that it is worthwhile to persist with working out with the health care team what provides the best outcomes.

The pain medications most frequently reported to be effective included cannabinoids, opioid‐containing medications, antimigraine, medications containing both paracetamol and NSAIDs, and sedatives (> 70% reported as resulting in much improvement, very much improvement or complete pain relief). Similarly, previous studies indicated substantial pain improvements following the use of opioids [8, 31], cannabinoids [8, 31, 53] and sedatives [31], despite variation in the method of pain measurements [31]. For NSAIDs and anticonvulsants, still 60%–70% were reported to result in much improvement, very much improvement or complete pain relief, this being 50%–60% for paracetamol, muscle relaxants and antidepressants. Thus, the over‐the‐counter medications most commonly used (i.e., paracetamol alone, NSAIDs alone) may not provide optimal pain relief in PwMS and may need to be supplemented with higher efficacy medications. The relatively higher perceived effectiveness for medications containing both paracetamol and NSAIDs, compared to paracetamol alone or NSAIDs alone, aligns with the principle of multimodal analgesia mentioned before. Variations in the effect of medications suggest the importance of a personalised approach to optimise pain relief in PwMS.

While the quantitative survey did not collect information on the pain types for which participants were using each medication, qualitative data indicated that some pain medications provided either substantial or moderate improvement for specific pain types, while others were effective only for a short duration. For example, opioids, cannabinoids and muscle relaxants were more commonly mentioned in relation to spasticity‐related pain. This aligns with findings from systematic reviews that have shown that the effectiveness of opioids [54] and cannabinoids [55] is higher when used for spasticity‐related pain. In contrast, the effectiveness of anticonvulsants and antidepressants on pain relief is more common when used for neuropathic pain [48, 56]. We also found that the effectiveness of some medications, including antidepressants and anticonvulsants, may be limited to a short time period only, with some comments, indicating that ‘they have life‐changing effects for a short time’. A recent review further indicated that opioid tolerance is a problem for longer term use, as it may lead to neuroadaptive changes requiring progressively higher doses to achieve the same level of pain relief [57]. However, in the quantitative data, we did not measure the effectiveness of pain medication by pain type or whether its effectiveness diminished over long‐term use in the quantitative data. Therefore, integrating qualitative and quantitative data offered a more comprehensive understanding of pain medication use and effectiveness by identifying differences in effectiveness based on pain type and showing that the effectiveness of some medications may diminish over time, insights that would not have been identified through quantitative analysis alone.

A total of 40.2% of PwMS who had pain regularly stopped using medications, with opioids containing medications, anticonvulsants, and NSAIDs being most frequently stopped. The main reasons for stopping were that participants did not like the side effects and that the medications were ineffective. The medication types most frequently stopped due to side effects, including sedatives, anticonvulsants, antidepressants, muscle relaxants and opioid‐containing medications. In contrast, paracetamol was most frequently stopped due to perceived ineffectiveness. Severe adverse effects were less common. Some focus group participants indicated they avoid the use of some or any medications due to concerns around side effects and dependence. It is important for health professionals to discuss this with their patients and to consider starting patients on low doses to improve tolerability.

The strengths of our study lie in its large sample size of PwMS for quantitative data. Additionally, the utilisation of a mixed‐methods approach enhanced the depth of the analysis, providing a nuanced understanding of medication usage and its effects on pain. Perceived effectiveness was measured only among those who were using pain medications at the time of the survey, but some may have been using some medications for some time. As mentioned above, this creates a self‐selection, also known as a ‘depletion of susceptible effect’ [58], which needs to be considered when interpreting that self‐reported data. For pain medications stopped in the past, there may be potential recall bias regarding the reasons for stopping. Those participating in the survey were slightly older compared to those who did not participate, while there was no difference in sex, disease duration and education level. We examined whether having a slightly older sample affected the key findings and found that there was no difference in terms of overall pain medication use and little difference in the perceived effectiveness of all medications. However, older participants were less likely to stop using medication, suggesting that we may have slightly underestimated the percentage of medications stopped. In the survey, we did not ask for which pain type they were using each medication, as this is difficult to self‐report. It is, however, useful to know the effectiveness of each medication type for the different pain types.

In conclusion, the results of this study indicate that pain medications are commonly used among PwMS, with the use of multimodal therapies frequently required. Side effects and ineffectiveness are often encountered; therefore, adjustments to treatment plans might be needed. Good communication between patients and their healthcare practitioners, as well as patient education on strategies to limit side effects, is key to effective pain control. Our findings suggest that effective pain control is achievable for most PwMS who have pain regularly.

## Author Contributions

The quantitative study was designed by Alice Saul, Kristen Lefever, L. Laura Laslett, V. Bruce Taylor and Ingrid van der Mei; the data collection was overseen by Ingrid van der Mei, and the data were cleaned and managed by Mohammed Suleiman Obsa, Alice Saul, Kristen Lefever and L. Laura Laslett. Kristen Lefever and V. Bruce Taylor provided clinical expertise in the data cleaning. The data analysis was performed by Mohammed Suleiman Obsa, guided by Alice Saul, L. Laura Laslett and Ingrid van der Mei. The qualitative study was designed by A. Julie Campbell and Ingrid van der Mei, and the data were collected by A. Julie Campbell. The qualitative data were analysed by Mohammed Suleiman Obsa, guided by A. Julie Campbell. Mohammed Suleiman Obsa had the primary role in preparing the manuscript, which was primarily edited by Alice Saul, A. Julie Campbell and Ingrid van der Mei. Later‐stage feedback was received from Kristen Lefever, L. Laura Laslett, Baye Dagnew and V. Bruce Taylor.

## Funding

This study was supported by MS Australia. Alice Saul, A. Julie Campbell, L. Laura Laslett and Ingrid van der Mei were supported by fellowships from MS Australia, and V. Bruce Taylor was supported by an NHMRC Investigator Grant. Mohammed Suleiman Obsa and Baye Dagnew were supported by the Tasmania Graduate Research Scholarship and Menzies Institute for Medical Research Top‐Up Scholarship. Open access publishing facilitated by University of Tasmania, as part of the Wiley ‐ University of Tasmania agreement via the Council of Australasian University Librarians.

## Disclosure

All authors were involved in the data interpretation and approved the final version of the manuscript and agreed to be accountable for all aspects of the work. The funder has not participated in the analysis and interpretation of data, the writing of the report, or the decision to submit the article for publication.

## Conflicts of Interest

The authors declare no conflicts of interest.

## Supporting Information

Additional supporting information can be found online in the Supporting Information section.

## Supporting information


**Supporting Information 1** Supporting Table 1: Good reporting of a mixed‐methods study (GRAMMS) checklist.

**Supporting Information 2** Supporting Table 2: Consolidated criteria for reporting qualitative studies (COREQ): 32‐item checklist.

**Supporting Information 3** Supporting Table 3: Supporting file 3. STROBE statement—checklist of items that should be included in reports of cross‐sectional studies.

## Data Availability

MS Australia owns the AMSLS data and is accessible to MS state organisations, researchers and other stakeholders seeking to benefit from this resource. Data can be accessed via the data request form https://www.msaustralia.org.au/amsls/researchers/.

## References

[1] Foley P. L. , Vesterinen H. M. , Laird B. J. et al., Prevalence and Natural History of Pain in Adults With Multiple Sclerosis: Systematic Review and Meta-Analysis, Pain. (2013) 154, no. 5, 632–642, 10.1016/j.pain.2012.12.002. 23318126

[2] Zhang Y. , Taylor B. V. , Simpson S.Jr. et al., Feelings of Depression, Pain and Walking Difficulties Have the Largest Impact on the Quality of Life of People With Multiple Sclerosis, Irrespective of Clinical Phenotype, Multiple Sclerosis. (2021) 27, no. 8, 1262–1275, 10.1177/1352458520958369. 32924841

[3] Murphy K. L. , Bethea J. R. , and Fischer R. , Neuropathic Pain in Multiple Sclerosis–Current Therapeutic Intervention and Future Treatment Perspectives, 2017, Exon Publications. 29261265

[4] Truini A. , Barbanti P. , Pozzilli C. , and Cruccu G. , A Mechanism-Based Classification of Pain in Multiple Sclerosis, Journal of Neurology. (2013) 260, no. 2, 351–367, 10.1007/s00415-012-6579-2. 22760942 PMC3566383

[5] Lo L. M. P. , Taylor B. V. , Winzenberg T. et al., Estimating the Relative Contribution of Comorbidities in Predicting Health-Related Quality of Life of People With Multiple Sclerosis, Journal of Neurology. (2021) 268, no. 2, 569–581, 10.1007/s00415-020-10195-w. 32880072

[6] Brola W. , Mitosek-Szewczyk K. , and Opara J. , Symptomatology and Pathogenesis of Different Types of Pain in Multiple Sclerosis, Neurologia i Neurochirurgia Polska. (2014) 48, no. 4, 272–279, 10.1016/j.pjnns.2014.07.009. 25168327

[7] Brichetto G. , Uccelli M. M. , Mancardi G. , and Solaro C. , Symptomatic Medication Use in Multiple Sclerosis, Multiple Sclerosis Journal. (2003) 9, no. 5, 458–460, 10.1191/1352458503ms957oa. 14582769

[8] Kratz A. L. , Whibley D. , Alschuler K. N. et al., Characterizing Chronic Pain Phenotypes in Multiple Sclerosis: A Nationwide Survey Study, Pain. (2021) 162, no. 5, 1426–1433, 10.1097/j.pain.0000000000002136. 33196577 PMC8054538

[9] Chisari C. G. , Sgarlata E. , Arena S. , D’Amico E. , Toscano S. , and Patti F. , An Update on the Pharmacological Management of Pain in Patients With Multiple Sclerosis, Expert Opinion on Pharmacotherapy. (2020) 21, no. 18, 2249–2263, 10.1080/14656566.2020.1757649. 32343626

[10] Dagnew B. , Campbell J. A. , Laslett L. L. et al., Understanding Pain Types and the Lived Experiences of Individuals With Multiple Sclerosis and Pain: A Mixed Methods Study, Multiple Sclerosis and Related Disorders. (2025) 104, 10.1016/j.msard.2025.106778. 41027275

[11] Breuer B. , Pappagallo M. , Knotkova H. , Guleyupoglu N. , Wallenstein S. , and Portenoy R. , A Randomized, Double-Blind, Placebo-Controlled, Two-Period, Crossover, Pilot Trial of Lamotrigine in Patients With Central Pain due to Multiple Sclerosis, Clinical Therapeutics. (2007) 29, no. 9, 2022–2030, 10.1016/j.clinthera.2007.09.023. 18035201

[12] Corey-Bloom J. , Wolfson T. , Gamst A. et al., Smoked Cannabis for Spasticity in Multiple Sclerosis: A Randomized, Placebo-Controlled Trial, Canadian Medical Association Journal. (2012) 184, no. 10, 1143–1150, 10.1503/cmaj.110837. 22586334 PMC3394820

[13] Rog D. J. , Nurmikko T. J. , and Young C. A. , Oromucosal delta9-Tetrahydrocannabinol/Cannabidiol for Neuropathic Pain Associated With Multiple Sclerosis: An Uncontrolled, Open-Label, 2-Year Extension Trial, Clinical Therapeutics. (2007) 29, no. 9, 2068–2079, 10.1016/j.clinthera.2007.09.013. 18035205

[14] Rossi S. , Mataluni G. , Codeca C. et al., Effects of Levetiracetam on Chronic Pain in Multiple Sclerosis: Results of a Pilot, Randomized, Placebo-Controlled Study, European Journal of Neurology. (2009) 16, no. 3, 360–366, 10.1111/j.1468-1331.2008.02496.x. 19364364

[15] Turcotte D. , Doupe M. , Torabi M. et al., Nabilone as an Adjunctive to Gabapentin for Multiple Sclerosis-Induced Neuropathic Pain: A Randomized Controlled Trial, Pain Medicine. (2015) 16, no. 1, 149–159, 10.1111/pme.12569. 25288189

[16] Vermersch P. and Trojano M. , Tetrahydrocannabinol: Cannabidiol Oromucosal Spray for Multiple Sclerosis-Related Resistant Spasticity in Daily Practice, European Neurology. (2016) 76, no. 5-6, 216–226, 10.1159/000449413. 27732980

[17] Carnero C. E. , López P. A. , Criniti J. et al., Use of Cannabis in Patients With Multiple Sclerosis From Argentina, Multiple Sclerosis and Related Disorders. (2021) 51. 10.1016/j.msard.2021.102932 33848817

[18] Giossi R. , Mercenari M. , Filippi M. et al., Unprescribed Cannabinoids and Multiple Sclerosis: A Multicenter, Cross-Sectional, Epidemiological Study in Lombardy, Italy, Journal of Neurology. (2024) 271, no. 11, 7186–7205, 10.1007/s00415-024-12472-4. 38844694 PMC11561032

[19] Chong M. S. , Wolff K. , Wise K. , Tanton C. , Winstock A. , and Silber E. , Cannabis Use in Patients With Multiple Sclerosis, Multiple Sclerosis. (2006) 12, no. 5, 646–651, 10.1177/1352458506070947. 17086912

[20] Carotenuto A. , Costabile T. , De L. M. et al., Predictors of Nabiximols (Sativex®) Discontinuation Over Long-Term Follow-Up: A Real-Life Study, Journal of Neurology. (2020) 267, no. 6, 1737–1743, 10.1007/s00415-020-09739-x. 32124041

[21] Marrie R. A. , Fisk J. D. , Walld R. et al., Prescription Opioid Use in Multiple Sclerosis, Journal of Neurology, Neurosurgery & Psychiatry. (2023) 94, no. 2, 167–169, 10.1136/jnnp-2022-329508. 36028309 PMC9872229

[22] Turner A. P. , Arewasikporn A. , Hawkins E. J. et al., Risk Factors for Chronic Prescription Opioid Use in Multiple Sclerosis, Archives of Physical Medicine and Rehabilitation. (2023) 104, no. 11, 1850–1856, 10.1016/j.apmr.2023.04.012. 37137460

[23] Fu X. , Wang Y. , Wang C. et al., A Mixed Treatment Comparison on Efficacy and Safety of Treatments for Spasticity Caused by Multiple Sclerosis: A Systematic Review and Network Meta-Analysis, Clinical Rehabilitation. (2018) 32, no. 6, 713–721, 10.1177/0269215517745348. 29582713

[24] Sartori A. , Dinoto A. , Stragapede L. et al., Nabiximols and Botulinum Toxin Injections for Patients With Multiple Sclerosis: Efficacy on Spasticity and Spasms in a Single-Centre Experience, Neurological Sciences. (2021) 42, no. 12, 5037–5043, 10.1007/s10072-021-05182-6. 33742336

[25] Arroyo G. R. , A Review of the Effects of Baclofen and of THC:CBD Oromucosal Spray on Spasticity-Related Walking Impairment in Multiple Sclerosis, Expert Review of Neurotherapeutics. (2018) 18, 785–791. 30235965 10.1080/14737175.2018.1510772

[26] Bethoux F. A. , Farrell R. , Checketts D. et al., A Randomized, Double-Blind, Placebo-Controlled Trial to Evaluate the Effect of Nabiximols Oromucosal Spray on Clinical Measures of Spasticity in Patients With Multiple Sclerosis, Multiple Sclerosis and Related Disorders. (2024) 89, 10.1016/j.msard.2024.105740. 39106541

[27] Mousavi P. , Emadzadeh M. , Karimikhoshnoudian B. et al., A Randomized Trial on Efficacy of Purified Cannabidiol on Spasticity in Multiple Sclerosis Patients With Gait Problems: First Report in Iran, Naunyn-Schmiedeberg’s Archives of Pharmacology. (2025) 398. 10.1007/s00210-025-04347-w 40498097

[28] Hansen J. S. , Hansen R. M. , Petersen T. et al., The Effect of Cannabis-Based Medicine on Neuropathic Pain and Spasticity in Patients With Multiple Sclerosis and Spinal Cord Injury: Study Protocol of a National Multicenter Double-Blinded, Placebo-Controlled Trial, Brain Sciences. (2021) 11, no. 9, 10.3390/brainsci11091212. PMC8465969 34573231

[29] Zertal A. , Alami Marrouni K. , Arbour N. et al., Efficacy of Cannabinoids Compared to the Current Standard Treatments on Symptom Relief in Persons With Multiple Sclerosis (CANSEP Trial): Study Protocol for a Randomized Clinical Trial, Frontiers in Neurology. (2024) 15, 10.3389/fneur.2024.1440678. PMC11303178 39114536

[30] Zajicek J. P. , Hobart J. C. , Slade A. , Barnes D. , and Mattison P. G. , Multiple Sclerosis and Extract of Cannabis: Results of the MUSEC Trial, Journal of Neurology Neurosurgery and Psychiatry. (2012) 83, no. 11, 1125–1132, 10.1136/jnnp-2012-302468. 22791906

[31] Ehde D. M. , Alschuler K. N. , Osborne T. L. , Hanley M. A. , Jensen M. P. , and Kraft G. H. , Utilization and Patients’ Perceptions of the Effectiveness of Pain Treatments in Multiple Sclerosis: A Cross-Sectional Survey, Disability and Health Journal. (2015) 8, no. 3, 452–456, 10.1016/j.dhjo.2015.03.001. 25899795 PMC4464976

[32] Heckman-Stone C. and Stone C. , Pain Management Techniques Used by Patients With Multiple Sclerosis, Journal of Pain. (2001) 2, no. 4, 205–208, 10.1054/jpai.2001.23133. 14622818

[33] Curry L. A. , Nembhard I. M. , and Bradley E. H. , Qualitative and Mixed Methods Provide Unique Contributions to Outcomes Research, Circulation. (2009) 119, no. 10, 1442–1452, 10.1161/circulationaha.107.742775. 19289649

[34] Dossett L. A. , Kaji A. H. , and Dimick J. B. , Practical Guide to Mixed Methods, JAMA Surgery. (2020) 155, no. 3, 254–255, 10.1001/jamasurg.2019.4388. 31995145

[35] O’Cathain A. , Murphy E. , and Nicholl J. , Good Reporting of a Mixed Methods Study (GRAMMS) Checklist, Journal of Health Services Research & Policy. (2008) 13, 92–98. 10.1258/jhsrp.2007.007074 18416914

[36] O’Brien B. C. , Harris I. B. , Beckman T. J. , Reed D. A. , and Cook D. A. , Standards for Reporting Qualitative Research: A Synthesis of Recommendations, Academic Medicine. (2014) 89, no. 9, 1245–1251, 10.1097/acm.0000000000000388. 24979285

[37] Tong A. , Sainsbury P. , and Craig J. , Consolidated Criteria for Reporting Qualitative Research (COREQ): A 32-Item Checklist for Interviews and Focus Groups, International Journal for Quality in Health Care. (2007) 19, no. 6, 349–357, 10.1093/intqhc/mzm042. 17872937

[38] Malterud K. , Siersma V. D. , and Guassora A. D. , Sample Size in Qualitative Interview Studies: Guided by Information Power, Qualitative Health Research. (2016) 26, no. 13, 1753–1760, 10.1177/1049732315617444. 26613970

[39] Ezzy D. , Qualitative Analysis, 2013, Routledge.

[40] Rice P. L. and Ezzy D. , Qualitative Research Methods: A Health Focus, 1999, 275, Oxford University Press.

[41] Campbell J. A. , Ezzy D. , Neil A. et al., A Qualitative Investigation of the Health Economic Impacts of Bariatric Surgery for Obesity and Implications for Improved Practice in Health Economics, Health Economics. (2018) 27, no. 8, 1300–1318, 10.1002/hec.3776. 29855095

[42] Glover J. and Tennant S. , Remote Areas Statistical Geography in Australia: Notes on the Accessibility/Remoteness Index for Australia (ARIA+ Version), 2003, Australian Bureau of Statistics.

[43] Daly D. and Sweeney B. , Patterns of Use and Effectiveness of Analgesics and Cannabinoids for Pain Relief Among Irish Patients With Multiple Sclerosis, Journal of the Neurological Sciences. (2021) 429, 10.1016/j.jns.2021.118115.

[44] Hugos C. L. , Joos S. , Sajeev N. , Norton J. , Samiee S. , and Cameron M. , One in Five (20%) People With Multiple Sclerosis Use Prescription Opioids (4926), Neurology. (2021) 96, no. 15_supplement, 10.1212/wnl.96.15_supplement.4926.

[45] Young J. , Amatya B. , Galea M. P. , and Khan F. , Chronic Pain in Multiple Sclerosis: A 10-Year Longitudinal Study, Scandinavian Journal of Pain. (2017) 16, no. 1, 198–203, 10.1016/j.sjpain.2017.04.070. 28850402

[46] Svendsen K. B. , Jensen T. S. , Overvad K. , Hansen H. J. , Koch-Henriksen N. , and Bach F. W. , Pain in Patients With Multiple Sclerosis: A Population-Based Study, Archives of Neurology. (2003) 60, no. 8, 1089–1094, 10.1001/archneur.60.8.1089. 12925364

[47] Hadjimichael O. , Kerns R. D. , Rizzo M. A. , Cutter G. , and Vollmer T. , Persistent Pain and Uncomfortable Sensations in Persons With Multiple Sclerosis, Pain. (2007) 127, no. 1, 35–41, 10.1016/j.pain.2006.07.015. 16949751

[48] Shkodina A. D. , Bardhan M. , Chopra H. et al., Pharmacological and Non-Pharmacological Approaches for the Management of Neuropathic Pain in Multiple Sclerosis, CNS Drugs. (2024) 38, no. 3, 1–20, 10.1007/s40263-024-01072-5. 38421578

[49] Solaro C. and Uccelli M. M. , Pharmacological Management of Pain in Patients With Multiple Sclerosis, Drugs. (2010) 70, 1245–1254. 20568832 10.2165/11537930-000000000-00000

[50] Brown J. D. , Potential Adverse Drug Events With Tetrahydrocannabinol (THC) due to Drug–Drug Interactions, Journal of Clinical Medicine. (2020) 9, no. 4, 10.3390/jcm9040919. PMC7231229 32230864

[51] Feketová S. , Waczulíková I. , Valkovič P. , and Mareš J. , Central Pain in Patients With Multiple Sclerosis, Journal of Multiple Sclerosis. (2017) 4.

[52] Solaro C. and Uccelli M. M. , Management of Pain in Multiple Sclerosis: A Pharmacological Approach, Nature Reviews Neurology. (2011) 7, no. 9, 519–527, 10.1038/nrneurol.2011.120. 21844896

[53] Zajicek J. , Fox P. , Sanders H. et al., Cannabinoids for Treatment of Spasticity and Other Symptoms Related to Multiple Sclerosis (CAMS Study): Multicentre Randomised Placebo-Controlled Trial, Lancet. (2003) 362, no. 9395, 1517–1526, 10.1016/s0140-6736(03)14738-1. 14615106

[54] Busse J. W. , Wang L. , Kamaleldin M. et al., Opioids for Chronic Noncancer Pain: A Systematic Review and Meta-Analysis, JAMA. (2018) 320, no. 23, 2448–2460, 10.1001/jama.2018.18472. 30561481 PMC6583638

[55] Filippini G. , Minozzi S. , Borrelli F. , Cinquini M. , and Dwan K. , Cannabis and Cannabinoids for Symptomatic Treatment for People With Multiple Sclerosis, Cochrane Database of Systematic Reviews. (2022) 5. 10.1002/14651858.CD013444.pub2 PMC9069991 35510826

[56] Murphy K. , Bethea J. , and Fischer R. , Multiple Sclerosis: Perspectives in Treatment and Pathogenesis: Neuropathic Pain in Multiple Sclerosis – Current Therapeutic Intervention and Future Treatment Perspectives, 2017, https://exonpublications.com/index.php/exon/article/view/153.

[57] Mercadante S. , Arcuri E. , and Santoni A. , Opioid-Induced Tolerance and Hyperalgesia, CNS Drugs. (2019) 33, no. 10, 943–955, 10.1007/s40263-019-00660-0. 31578704

[58] Moride Y. and Abenhaim L. , Evidence of the Depletion of Susceptibles Effect in Non-Experimental Pharmacoepidemiologic Research, Journal of Clinical Epidemiology. (1994) 47, 731–737. 7722586 10.1016/0895-4356(94)90170-8

