# Supplementary material for: Pain Medication Use and Perceived Effectiveness in People With Multiple Sclerosis: A Mixed‐Methods Study
Source: Pain Res Manag. 2026 Jul 29;2026:9981770. doi: 10.1155/prm/9981770 (PMC13419084; doi:10.1155/prm/9981770)
Supplement: Supplementary file 1 — Supporting Information 1 Supporting Table 1: Good reporting of a mixed‐methods study (GRAMMS) checklist. [file PRM-2026-9981770-s002.docx]

| **Supplementary file 1. Good Reporting of A Mixed Methods Study (GRAMMS) checklist** | |
| --- | --- |
| **GRAMMS Item** | **Page (Pg) number** |
| 1. Justification to use a mixed methods approach to the research question | Methods: Pg 5 |
| 1. Articulation of the design in terms of purpose, priority, and sequence of methods | Methods: Pg 6-10 |
| 1. Describe each method in terms of sampling, data collection and analysis | Methods: Quantitative Data Collection & Quantitative Data Analysis: Pg 6-8  Qualitative Data Collection & Qualitative Data Analysis: Pg 7-9 |
| 1. Delineate where and how integration occurs and who has participated in it | Methods: Pg 5-6, 8-9,  Results: Pg 12  Discussion: Pg 16, 18-19 |
| 1. Describe any limitation of one method associated with the presence of another | Discussion: pg 18-19 |
| 1. Describe insights gained from mixing or integrating methods | Discussion: Pg 16, 18-19 |
| Reference: O'Cathain A, Murphy E, Nicholl J. The quality of mixed methods studies in health services research. J Health Serv Res Policy. 2008;13: 92-98. | |
